# Evaluating a multidimensional strategy to improve the professional self-care of occupational therapists working with people with life limiting illness

**DOI:** 10.1186/s12904-020-00695-x

**Published:** 2021-01-04

**Authors:** Courtney Apostol, Kathryn Cranwell, Danielle Hitch

**Affiliations:** 1 Occupational Therapy, Western Health, Sunshine, Australia; 2 Occupational Therapy, Deakin University, Geelong, Australia; 3 Allied Health, Western Health, Sunshine Hospital, 176 Furlong Road, St, Albans, Victoria 3021 Australia

**Keywords:** Occupational therapy, Life limiting conditions, Professional self-care, Resilience

## Abstract

**Background:**

The term ‘life limiting conditions’ refers to premature death following decline from chronic conditions, which is a common circumstance in which occupational therapists work with people at the end of life. The challenges for clinicians of working with these patients have long been recognised, and may have a significant impact on their professional self-care. This study aimed to evaluate a multidimensional workplace strategy to improve the professional self-care of occupational therapists working with people living with a life limiting condition.

**Methods:**

A pre and post mixed methods survey approach were utilised, with baseline data collection prior to the implementation of a multidimensional workplace strategy. The strategy included professional resilience education, targeted supervision prompts, changes to departmental culture and the promotion of self-care services across multiple organisational levels. Follow up data collection was undertaken after the strategy had been in place for 2 years. Quantitative data were analysed descriptively, while qualitative data were subjected to thematic analysis.

**Results:**

One hundred three occupational therapists responded (*n* = 55 pre, *n* = 48 post) across multiple service settings. Complex emotional responses and lived experiences were identified by participants working with patients with life limiting conditions, which were not influenced by the workplace strategy. Working with these patients was acknowledged to challenge the traditional focus of occupational therapy on rehabilitation and recovery. Participants were confident about their ability to access self-care support, and supervision emerged as a key medium. While the strategy increased the proportion of occupational therapists undertaking targeted training, around half identified ongoing unmet need around professional self-care with this patient group. Demographic factors (e.g. practice setting, years of experience) also had a significant impact on the experience and needs of participants.

**Conclusions:**

The multidimensional workplace strategy resulted in some improvements in professional self-care for occupational therapists, particularly around their use of supervision and awareness of available support resources. However, it did not impact upon their lived experience of working with people with life limiting conditions, and there remain significant gaps in our knowledge of support strategies for self-care of occupational therapist working with this patient group.

## Background

Care for people in the final weeks of their life is referred to as palliative care, end of life care, terminal care, and/or advanced care. While the term ‘life threatening illness’ is used by the World Health Organisation [1], this is more usually associated with conditions seen in acute and subacute public health services in Australia. The Australian National Palliative Care Strategy uses the term ‘life limiting condition’ (LLC), which refers to premature death following long term decline from chronic conditions [2] and is the preferred terminology in the clinical context of this study.

In Australia, palliative care is delivered by diverse health services from primary to tertiary care, not all of whom exclusively see patients experiencing LLCs [3]. Occupational therapists may offer services to people with LLCs in multiple service settings, including acute services, community services and aged care [4]. Approximately 40% of Australian occupational therapists work with people with LLCs in inpatient or hospice settings, and previous research has found near 80% of people die within hospital or residential care settings [5, 6].

The role of occupational therapy with people experiencing LLCs is less well defined than other patient populations, however it broadly aims to maintain the best possible functional performance, and support symptomatic relief and quality of life [7, 8]. A recent study with patients and caregivers highlighted the benefit of occupational therapy interventions, even if they increased pain and/or fatigue in the short term [9].

Despite the potential impact of these tasks, many occupational therapists believe the profession was not being used to its full potential with people with LLCs [10], and there are also concerns about a lack of relevant education and development for this workforce. Evidence around education and training requirements in this area of practice is slowly increasing [8], and free online training provided by the Australian Healthcare and Hospitals Association has been incorporated into some pre-registration courses [11]. However, many have called for both the frequency and scope of education and training to be increased [10, 12–15].

### Professional self-care for clinicians working with people with life limiting conditions

The ability of clinicians to maintain their own health within their working lives is important to sustainable wellbeing, particularly when working with people with LLCs. Professional self-care may be conceived as a meaningful occupation that can support healthy survival in the clinicians professional role [16]. According to the Pan Occupational Paradigm, meaningful occupations include being, becoming, belonging and doing [17]. For professional self-care, doing refers to the methods and mediums utilized, while being refers to a clinician’s sense of their personal experience and capacity. Becoming relates to the growth and development clinicians make in their self-care, while belonging describes the connections clinicians have with people, places, and communities which impact upon their professional self-care. This paradigm provided a guiding framework for this study to both embed the findings in the disciplinary context of occupational therapy, and describe the key features of self-care across diverse service settings.

Clinician self-care within medicine and nursing is well recognised as a key issue when working with patients with LLCs [18–20], however evidence from these disciplines may not be generalizable to occupational therapy [13]. Sanchez-Reilly et al., [19] highlighted that ineffective self-care can compromise clinicians’ personal well-being, leading to burnout, distress, compassion fatigue, and poor clinical decisions which may adversely affect patient care. In a study of burnout experiences for occupational therapists across diverse settings [21], work-related stress was found to lead to job dissatisfaction, low-organizational commitment, absenteeism, and high turnover. Occupational therapists with higher emotional exhaustion also reported less use of coping strategies and poorer physical self-care. Similarly, a study of rehabilitation professionals (including occupational therapists) [22] found that lower scores on subscales related to control, management support, relationships, roles and change were associated with a higher risk of burnout.

While negative wellbeing outcomes are mentioned in previous occupational therapy studies with people with LLCs [5], it is rarely the direct focus of research. A qualitative study of occupational therapists in hospice services [23] described how their sense of fulfillment enabled the clinicians to sustain their role and effectively practice therapeutically. The methods of self-care employed by these clinicians included leisure occupations, spending time with friends and family, spiritual practices, part time work, flexible work schedules and participating in workplace support programs. No other current occupational therapy research has addressed therapist self-care when working with this patient group in depth, other than to urge more development. However, a systematic review of psychosocial interventions to improve the wellbeing of staff who work in the palliative setting [24] found these interventions tended to comprise multiple elements (including relaxation, education, support and cognitive training), and target stress, fatigue, burnout, depression and satisfaction. However, it is unclear whether these findings are also relevant to service settings other than those specifically designated as ‘palliative’.

### Aim of study

This study aimed to evaluate a multidimensional workplace strategy to improve the professional self-care of occupational therapy clinicians working with people living with a life limiting condition. This study also sought to;

- Describe the being of clinicians, in regards to their demographic characteristics, emotional experiences and self-perception of self-care capacity
- Describe the becoming and belonging of clinicians, in regards to their understanding and awareness of resources available to assist with professional self-care
- Describe the doing of clinicians, by evaluating the impact of the multidimensional workplace strategy on wellbeing

## Methods

### Design

This study utilised a pre and post mixed methods survey approach. Ethics approval was received from the health service (QA2014_118). The study was initiated in response to practice experiences that suggested a pervasive lack of awareness around supports available for clinicians experiencing grief and loss when working with people with LLCs. Responses to and support around these experiences were individually based, and were not consistently available across the service despite recognition that some parts of the workforce were exposed to death and dying more frequently. The service was concerned that insufficient supports for the occupational therapy workforce could have a negative impact on staff retention, job satisfaction, mental health, and work- life balance.

### Setting

This study occurred in a large public health service located in a metropolitan location in Australia. The service consists of four acute/subacute campuses, and a diverse range of community based services. It serves a local community of over 800,000 people, which is among one of the most culturally diverse and socio-economically disadvantaged in Australia. The occupational therapy department includes 76 clinicians, supported by 13 allied health assistants, working across three main sites, providing therapy across multiple clinical cohorts including acute services, aged care, community clinics (e.g. plastics, paediatrics, aged care assessment services), and rehabilitation (both inpatient and community based). Participants in this study came from all of these service settings.

### Procedure

The study commenced with presentations about its aims and methodology to occupational therapy clinicians at team meetings, which also included written information and an opportunity to ask questions. All clinicians were then invited to participate in a mixed methods survey by email, hosted on the Survey Monkey™ platform, which was undertaken over a 2 week period in February 2015. Consent was implied if clinicians submitted their responses for analysis. The survey was developed based on a previous tool used in a study with nurses experiencing grief while working in intensive care [20], supplemented by a literature review of existing evidence. The survey consisted of 16 questions (8 quantitative and 8 qualitative), arranged into five themes – demographics, exposure, impact, strategies, and resources (please see Additional file 1). The same survey was re-administered over a 2 week period in May 2016, after the implementation of the multidimensional workplace strategy. While matched responses provide a more rigorous approach to pre-post methods, the high level of turnover in allied health workforces [25] often mitigates against this approach and in a small workforce such as occupational therapy there is an increased risk of inadvertent identification. Responses are therefore not matched in this study.

### Multidimensional workplace strategy

The multidimensional workplace strategy evaluated in this study was developed following initial data collection, to ensure it was relevant to the needs and preferences of the clinicians. Strategies proposed to be supportive of clinician professional self-care when working with people with LLCs included; i) Professional resilience education (e.g. stress management and self-care in-services), ii) supervision prompts, iii) changes to department culture and iv) promotion of self-care services. Based on the assumption that similar needs may also be present in other disciplines and practice settings, the strategy was implemented across three organisational levels: Occupational Therapy Department, Allied Health Directorate, and Organization wide. All components of the strategy took place in the workplace, during working hours. Table 1 below provides an overview of the multidimensional strategy, and each component of which will be described in further detail below. Further specific content information for each component is also available upon request from the author.

**Table 1:** Multidimensional Workplace Strategy

Level / Strategy	Occupational Therapy Department	Allied Health Directorate	Organization Wide
Professional Resilience Education (e.g. stress management and self-care in-services)	Capabilities Committee: Facilitated 1.5 h workshop “Roads to Resilience: Self-care Strategies for Occupational Therapists”.	Allied Health Profile & Culture Committee: Wellbeing Week, including mindfulness workshop and other activities	
Supervision Prompts	“Self care” placed on the supervision record form, as a simple reminder for regular discussion. The term ‘self care’ was subsequently included in the ‘Attitudes, Behaviour & Culture’ section of a newly developed Allied Health wide supervision record.		
Changes to Departmental Culture	Culture Committee: Promotion of RUOK Day Departmental newsletter features Professional resilience as standing agenda item Senior Leadership Group: Monitoring cultures across campuses		Health and Wellbeing Committee: 2015–2017 Health and Wellbeing Plan
Promoting Self-Care Services		Health and Wellbeing Committee: Workforce survey and resulting promotion of services and strategies	

#### Occupational therapy department

Three elements in the department were selected to address self-care strategies identified by clinicians; the Culture Committee, the Capabilities Committee and the Senior Leadership Group. The Culture Committee (which meets every 4 weeks) is responsible for leading and driving the review and strategic development of Occupational Therapy culture through promotion, professional integrity, and occupation based practice. This committee promoted departmental initiatives such as “R U OK” Day (An Australian non-profit suicide prevention organisation that advocates for people to freely discuss mental health); Self-care features in the bi-yearly Department Newsletter; and a commitment to professional resilience as a standing committee agenda item. The Capabilities Committee (which meets every 6 weeks) assists clinicians and students to identify and access education and training needs, which develop their capability to provide best care for patients. This committee facilitated a department wide workshop called “Roads to Resilience: Self-care strategies for Occupational Therapists”, presented by a leading researcher in this field [26]. Finally, the Senior Leadership Group (which also meets monthly) undertook the monitoring and maintaining of departmental culture across all campuses, as part of their usual oversight of service delivery and service planning.

#### Allied health directorate

The monthly Allied Health Profile & Culture Committee included three occupational therapy representatives including the Occupational Therapy Manager as chair, and aims to both develop a shared and cohesive Allied Health identity, and strengthen commitment of working together to continually improve the patient experience. This committee hosted a “Wellbeing Week” as part of the strategy implementation which included a series of staff wellbeing activities (shared lunches, yoga, lunchtime walks and exercise), and a mindfulness forum led by an expert in this area [27].

#### Organisation wide

An organizational wide Health and Wellbeing Committee was established, which meets monthly and includes an occupational therapy advisor. The committee established a health and wellbeing plan to guide occupational health and wellbeing initiatives (including clinician self-care). It also completed an organization-wide survey in December 2015 to identify key areas for clinicians and volunteers in regards to improving their health and wellbeing at work. The top ten needs identified from this survey were promoted widely, including an explicit discussion of what the service was doing to address them.

### Data analysis

Sample size calculations were not undertaken for this study, as the occupational therapy workforce at the health service already defined the target sample. However, response rates were calculated to provide an indication of the sufficiency of the sample in representing the experiences of occupational therapists at this health service. The use of a purposive sample was appropriate given that the intervention was only delivered at the study site. However it did introduce the possibility of selection bias, possibly due to self selection, non-response and/or attrition from the first survey.

All de-identified data from both surveys were entered into an Excel worksheet (Version 14.7.7), which included both quantitative and qualitative data. Quantitative data were then exported to SPSS Version 25 [28], and initially analysed descriptively. Chi-square analysis was also used to explore differences between demographic groups and over time. All qualitative data were subjected to independent coding by two researchers (CA, KC) before thematic analysis was finalised by collaboration [29]. A third researcher (DH) then reviewed this analysis, and any instances of disagreement were reconciled to establish the final thematic findings.

The Pan Occupational Paradigm [17] provided a framework for the final, integrated phase of analysis, which synthesised the quantitative and qualitative findings in response to the study aims and objectives. Both forms of data were compared to each other for each aspect of meaningful occupation – being, becoming, belonging and doing – and analysed for incidences of consonance and dissonance [30] as a basis for final reporting.

## Results

A similar proportion of the occupational therapy workforce responded to each survey, with 55 completing the baseline survey (approximate response rate – 73%) and 48 completing the second survey (approximate response rate – 64%). There were no differences in the pre intervention and post intervention samples in regards to grades (i.e. professional level), practice experience, practice setting, or experience with LLCs (either overall or in the past month). The clinical characteristics of participants are shown below in Table 2, and clinicians in aged care services and community settings were found to have significantly more practice experience than those in other practice settings [*X*^2^ (9, *N* = 103) = 22.95, *p* = .01].

**Table 2:** Study Participant Characteristics and Survey Scores

	2015 Survey			2016 Survey		
		n	%		n	%
Grades	AHA	3	5%	AHA	5	10%
	Grade 1	21	38%	Grade 1	11	23%
	Grade 2	24	44%	Grade 2	21	44%
	Grade 3	7	13%	Grade 3	11	23%
Practice Setting	Acute	18	33%	Acute	18	38%
	Rehabilitation	14	25%	Rehabilitation	10	21%
	Aged Care	8	15%	Aged Care	9	19%
	Community	15	27%	Community	11	23%
Practice Experience	0–3 years	15	27%	0–3 years	13	27%
	4–6 years	14	25%	4–6 years	7	15%
	7–10 years	13	24%	7–10 years	15	31%
	> 10 years	13	24%	> 10 years	13	27%
Previous experience with life limiting conditions	Yes	33	60%	Yes	33	69%
	No	22	40%	No	15	31%

Note: *AHA* Allied Health Assistants

### Being: demographic characteristics, emotional experiences and self-perception of self-care capacity

**Fig. 1:**
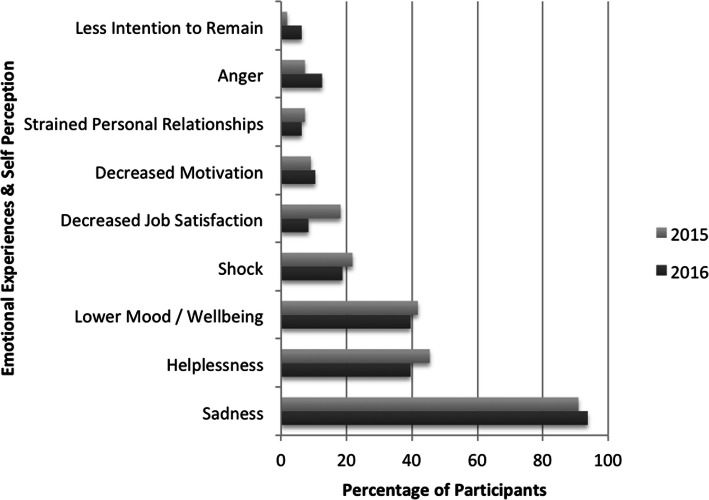
Percentage of Respondents Identifying Lived Experiences

Clinicians were asked about their experience of emotional responses (sadness, anger, shock, helplessness) and other lived experiences (low mood, decreased job satisfaction, decreased motivation, strain on personal relationships and intention to continue in current job / position) when providing therapy to people with LLCs, as shown in Fig. 1 below. The proportions of clinicians reporting these emotions and lived experiences were very similar across both surveys, with no statistically significant differences found (See Additional file 2).

Respondents indicated their experiences of working with people with LLCs consistently provoked feelings of sadness, helplessness and/or low mood. However, the emotional responses of shock and anger were less prevalent, as were the other lived experiences specified. Comparable numbers of respondents reported not experiencing any of the identified emotions (2015–7.27%, 2016–4.17%), and around half reported not experiencing any of the other lived experiences when working with people with LLCs (2015–46.25%, 2016–50.00%).

While there were no significant changes over time, significant differences were identified in regards to low mood and practice setting, with rehabilitation clinicians more likely to report low mood [96.77% Rehabilitation, 88.16% Other clinicians, *p* = .02]. Clinicians with between 3 and 5 years clinical experience were also significantly more likely to identify shock as an emotional response, than those in other career phases [47.62% 3–5 years, 13.41% Other Clinicians, *p* = .01].

Other emotions identified qualitatively in the surveys reflected negative, positive and neutral responses to working with people with LLCs. The most commonly reported emotion was frustration or stress, which was often in result of clinicians feeling they are not able to provide the level of care and support they would wish; *“Worry about not being afforded time to complete timely follow-up”*. These experiences were also related to feelings of guilt and incompetence if clinicians felt they were *‘not able to facilitate the outcomes the patients have wanted’*. Other negative emotions identified included shock and feeling ‘drained’. The traditional focus of occupational therapy on treatment and rehabilitation (rather than compensation and maintenance) was also identified as a potential dilemma; *“This can often make us feel uncomfortable as it is a complete change to that of how we normally respond to our clients and conversation around this can often be difficult”.*

Positive emotions were also identified, and were once again related to the perceived quality of care that clinicians could offer; “*Happiness, given patients and families are happy with engagement and they have reached the point of satisfaction and acceptance*”. The ability to provide meaningful support to patients at the end of their lives, and make a difference to their quality of life, were also related to feelings of joy and relief. Some respondents also reported feeling particularly motivated to work with people with LLCs. The experience of working with these patients also attracted some neutral emotional terms, such as ‘challenging’ and ‘eye opening’. While some clinicians experienced pity when working with this population, others used the more culturally prevalent term sympathy in their comments.

Some changes over time in regard to other lived experiences were indicated in the qualitative comments. Many clinicians initially reported ‘avoidant’ perceptions that sought to evade the need to work with people with LLCs; “*Less likely to want a rotation in high palliative patient caseload*”. However, there were fewer of these responses in the post-survey data. Comments from clinicians after the implementation of the multidimensional workplace strategy described more experiences of reconciliation to, and personal reflection about the death of patients; *“She had her journey in life and was not my place to be involved with that aspect of her journey”, “More concerned for your own health and your loved ones, more so heightened sense of your own mortality”.*

### Becoming and belonging - understanding and awareness of resources available to assist with professional self-care

**Fig. 2:**
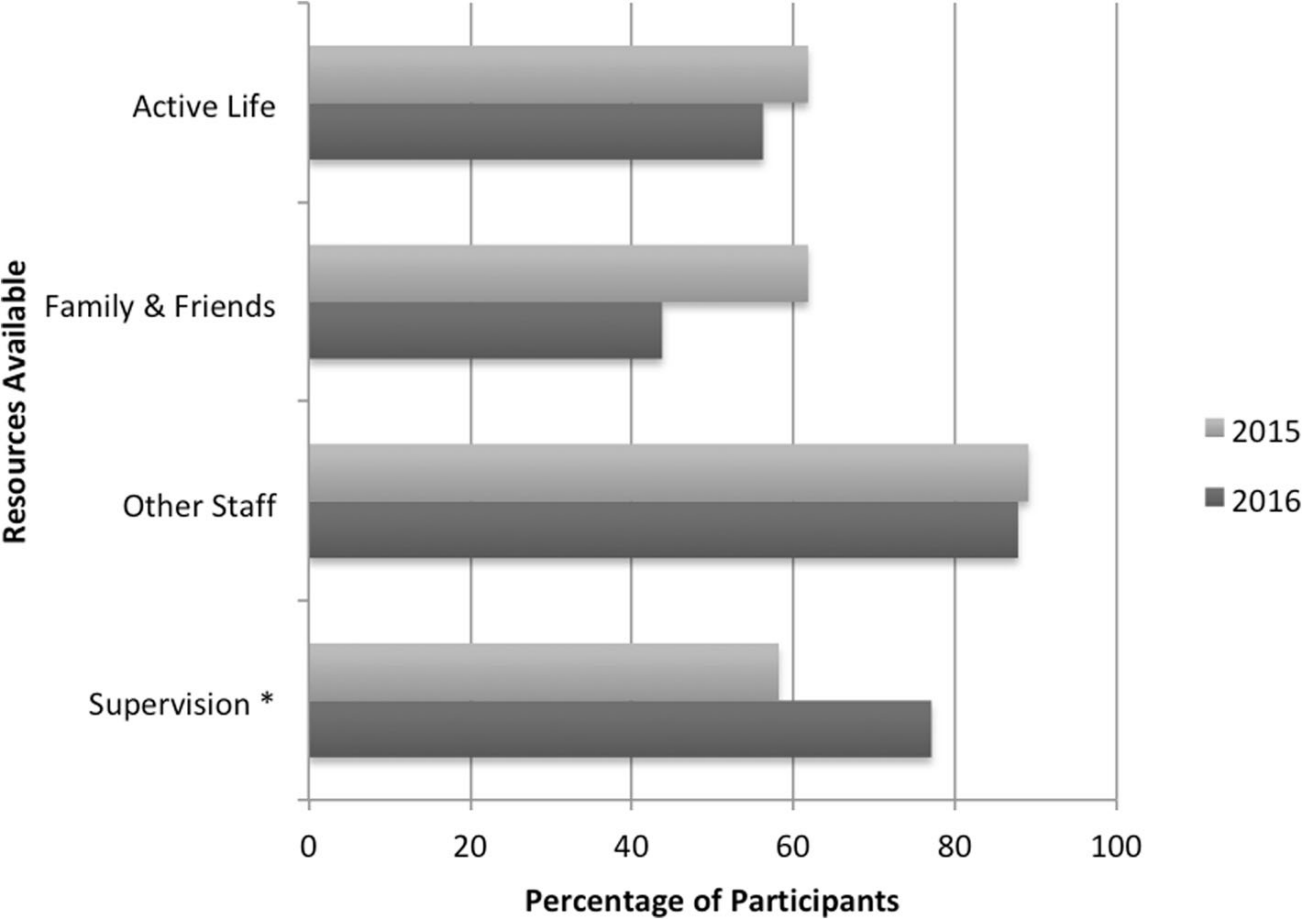
Percentage of Respondents Identifying Available Supports (* = Significant Change)

As shown below in Fig. 2, clinicians were asked about their experience of four specific sources of support (supervision, family and friends, leading an active life and other staff) when working with people with LLCs. The majority of respondents identified more than one support as personally relevant, with gaining support from other staff, and supervision prevalent.

While there were no significant differences over time for many of these resources, clinicians were significantly more likely to identify supervision as a support for self-care by the time of the second survey [58.18% 2015, 77.08% 2016, *p* = 0.04]. Practice setting did not have a significant influence on the identification of supports apart from seeking support from family and friends, with junior clinicians significantly more likely to identify this as a strategy [50.00% Junior clinicians, 21.57% Other clinicians, *p* = 0.02]. This was also reflected in an analysis of years of experience, where clinicians with six or less years of experience were significantly more likely to seek support from family and friends [40.54% < 6 years experience, 28.79% Other clinicians *p* = 0.04].

There was generally some level of awareness of support for professional self-care amongst respondents, with only one clinician in each survey stating they were not aware of any. While there was an increase in the number of respondents accessing some form of self-care service in relation to their professional roles with people with LLCs over time (2015–18.18%, 2016–25.00%), this was not statistically significant, *p* = 0.62.

Supervision was identified consistently as a support in qualitative comments, as was the workplace employee assistance program. While the majority of respondents reported they regularly received support for professional self-care during supervision, some participants expressed reservations; *“it can certainly be dependent on the supervisory relationship”*. However, comments indicated there was less engagement with the employee assistance program; *“[service name] has an employee assistance program. I have never used it and I very rarely remember to tell others about it*”. Another respondent identified this resource, but said; “*I doubt I would realistically even access this*”. In the baseline survey, participants also identified peer support or debriefing, professional resilience training and individual access to general practitioner mental health care plans in the initial survey. However, in the second survey professional resilience education and other professional development or training became more prominent.

**Fig. 3:**
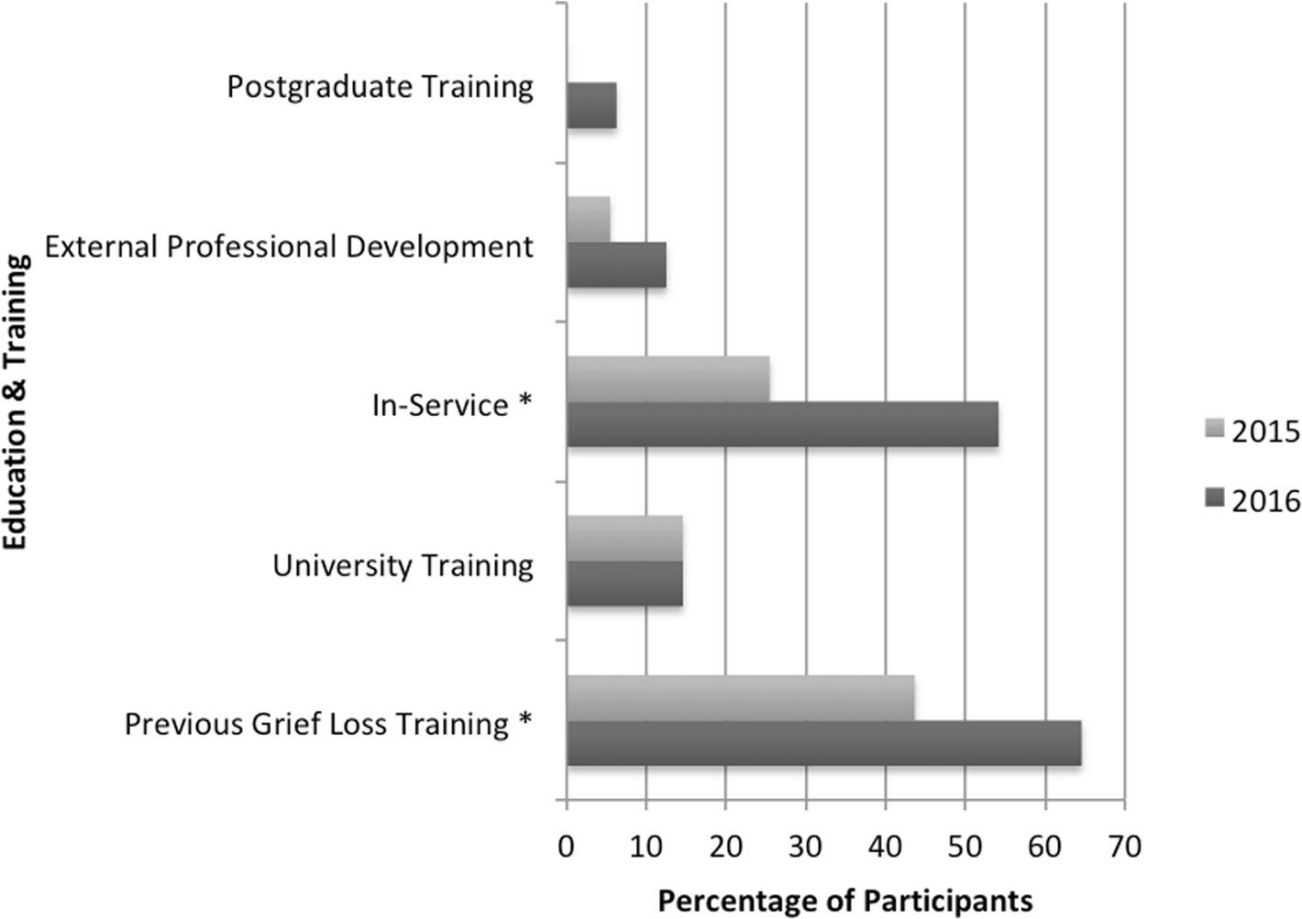
Percentage of Respondents Identifying Professional Development (* = Significant Change)

As shown below in Fig. 3, five forms of professional development relevant to professional self-care (post graduate training, external professional development, in-service, university training, previous grief and loss training) when working with people with LLCs were identified. Not all respondents reported attending this form of support, with workplace learning (i.e. in-services) and previous grief and loss training the most frequently identified.

The second cohort were significantly more likely to state they have previously received training in grief and loss [43.64% 2015, 64.58% 2016, *p* = .03], and to have participated in in-service training [25.45% 2015, 54.17% 2016, *p* = .01]. Clinicians at higher grades were also significantly more likely to identify previous grief and loss training than junior clinicians [77.78% Senior clinicians, 48.81% Other clinicians, *p* = .05], and to identify external professional development opportunities [21.43% Senior clinicians, 3.53% Other clinicians, *p* = .02]. Very few qualitative comments were received regarding professional development, most of which identified courses or modules provided by external providers.

Other, more personalised resources for professional self-care were also highlighted by some participants, demonstrating that respondents used resources both within the workplace and beyond; “*singing in the [service] Choir, occasional glass of wine at end of rough week!*” In the initial survey, participant’s highlighted personal strategies such as mindfulness and relaxation, however self reflection was identified as a theme in qualitative responses to the second survey (possibly in response to the workshops offered in the workplace intervention). Overall, a significant majority of respondents reported feeling confident in their ability to access resources to support personal self-care in both surveys (81.81% 2015, 91.67% 2016, *p* = 0.55).

### Doing - impact of implemented strategies on professional self-care for the workforce as a whole

**Fig. 4:**
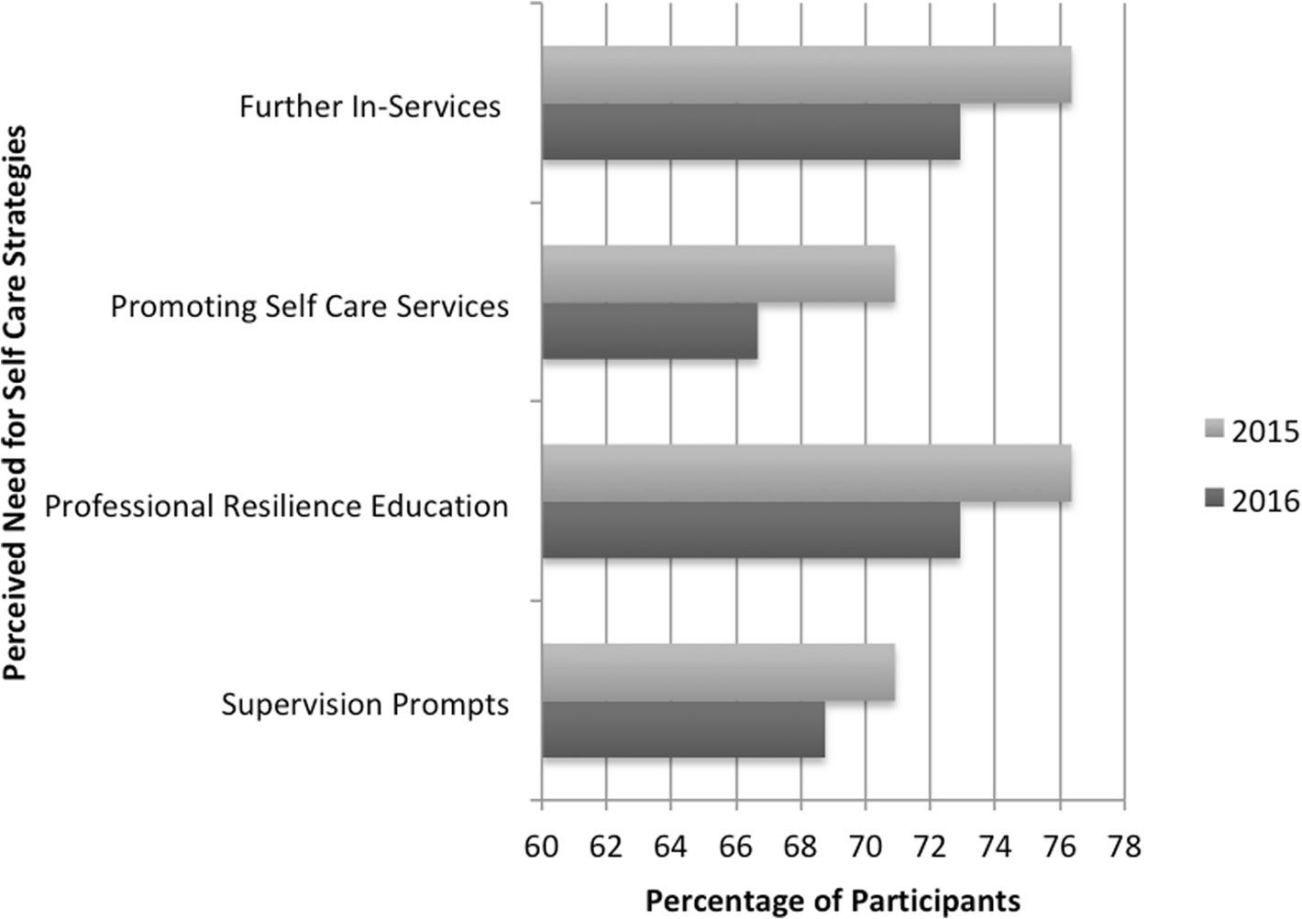
Percentage of Respondents Expressing a Need for Identified Strategies

The perceived need for three of the workplace strategies subsequently implemented was measured pre and post implementation. Changes to department culture were not directly measured, as it was assumed to be an emergent outcome of the implementation of these strategies. As shown below in Fig. 4, the majority of respondents in both surveys consistently expressed a need for these strategies to support their professional self-care.

Around half of the respondents in each survey (2015–49.09%, 2016–52.08%) stated they had further professional development needs in regards to professional self-care when working with people with LLCs. While not addressed in the quantitative questions, organizational culture was a consistent theme within the qualitative comments. Responses to the initial survey indicated respondents wanted greater resources within the organization, including references to the culture of the occupational therapy department; “*Changes to culture re: talking about and developing strategies to manage self-care in the workplace, protected time for these discussions and opportunities to share experiences and provide support to one another/discuss resources available*.” There were also comments related to the existing culture, which was perceived to insufficiently support professional self-care when working with these patients; *“It just feels like the expectation is often to continue on as if nothing has happened. Although it can be a very sad process, there is opportunity for satisfaction in the work – knowing that you are making a difference at end stage”.* However, there was also recognition that professional self-care needed a sustained focus to effect cultural change over time; *“With increasing emphasis on throughput and productivity, discussions and support networks for staff need to be prioritised and maintained. It is not always easy for staff to prioritise this due to high workload and personal expectations, so this needs to be modelled across different grades and supported/prompted by management”*.

By the time of the second survey, respondents had shifted their focus toward ‘external’ resources including further professional development, attending professional special interest groups and external courses. Consolidation of the multidimensional workplace strategies already implemented was also highlighted, with reference to sustaining these practices in an evolving workforce; “*A resource outlining key strategies from the recent resilience training would be great to refer back to - as new staff come through, it would be great to be able to have a record so that a more evidence-based/fuller range of strategies can be provided*”. However, there were also some indications of cultural changes at the organization, including a shift in perceptions around professional behavior when working with people with LLCs; *“It is important to know – it is ok to feel sad (don’t always need to be brave)”*.

## Discussion

The findings of this study have provided an insight into the impact of a multidimensional workplace strategy on the being, becoming, belonging and doing of professional self-care for occupational therapists working with people living with a life limiting condition. While many of these findings align with and complement previous research, some are novel and indicate new areas for investigation and development.

The findings of this study clearly illustrated the complex and personalised nature of emotional responses and other lived experiences around working with people with LLCs. There were no significant changes in these experiences during the implementation of the multidimensional workplace strategy, but this was not an aim of the strategy in any case. Similarly complex responses were also identified in a qualitative study with eight occupational therapists working with people with LLCs in the United States [31], who described experiences of satisfaction, hardships and difficulties, coping, spirituality and growth. Previous occupational therapy researchers have suggested that emotions are heightened when working with people with LLCs, particularly due to additional time pressures [31–34]. However, respondents in this study did not identify spirituality as a key aspect of their lived experience, despite it being identified in previous research within occupational therapy [31, 35].

Emotional responses were much more prevalent for respondents than other lived experiences (particularly those related to job perceptions). While the service was initially concerned about the impact of insufficient supports for professional self-care on retention, job satisfaction, mental health, and work life balance, they were not reported to be a major issue by respondents in this study. Similar findings have also been found in other research [5], where despite the challenges of working with people with LLCs the majority of occupational therapists intended to continue in their roles and reported relatively high levels of job satisfaction.

While shock and anger were less prevalent in this sample, related emotions such as frustration and stress were identified consistently within the qualitative comments. Stress and burnout in palliative care has been found to frequently be related to professional issues, but is not significantly more prevalent than rates found in other service settings [36]. The source of frustration and stress for occupational therapists in this study was attributed to feelings of not providing best care for people with LLCs, which is also similar to the experiences of loss of control and uncertainty reported by other occupational therapists working with similar patient cohorts [5, 10, 31, 37].

Respondents in this study (and others) also highlighted that working with these patients challenges the traditional focus in occupational therapy (and indeed healthcare) on therapy and rehabilitation [23, 31]. Functional maintenance and symptom management are generally perceived as subordinate to recovery or cure, however these support aspects of quality of life which are a priority to people at the end of life [38]. Theoretical approaches now beginning to emerge in occupational therapy [39, 40] may assist the discipline to resolve this perceived dilemma, as will increased awareness of what it can contribute to this population of patients [10, 32, 33].

In regards to seeking support for professional self-care, the majority of respondents expressed confidence in being able to access relevant resources. Along with peer support from other staff, supervision emerged as a key support for professional self-care, being identified by respondents significantly more frequently by the end of the implementation period. Supervision has previously been identified as an element of professional self-care in general healthcare [41], with the need for facilitation of deep reflection emphasized. It has also been highlighted as a key professional self-care strategy for nurses in palliative settings [42], however Edmonds et al. [43] acknowledge the best way to apply this strategy to enable professional self-care remains unclear. The findings in this study indicate that supervision for professional self-care is also relevant to occupational therapists working with people with LLCs more broadly, indicating further research in this area is indicated.

However, respondents were not so engaged with the workplace support program. While employee assistance programs have been shown to have positive outcomes for both employees and employers [44], previous research has shown there may be some stigma attached to attending [45] and professional self-care is not a prevalent presentation problem in these services [46]. The role of employee assistance programs in supporting professional self-care therefore remains poorly defined.

In regards to support for knowledge and skill building, workplace learning remained the predominant source for the respondents in this study. The significant increase in respondents stating they had previously received grief and loss, or in-service, training in the second survey is indicative of the impact of the multidimensional workplace strategy over this time. However, relatively few respondents (< 20%) identified their undergraduate training supporting their capacity for professional self-care when working with people with LLCs, which has also been flagged as a capacity issue by other authors [15, 23]. As highlighted by Hammill [8], there is potential to expand the professional development opportunities for occupational therapists beyond those offered in this workplace strategy to include clinical scenarios, face-to-face contact with clients, and additional workshops and seminars around topics relevant to professional self-care such as the occupational therapy role, disease trajectory, and communication. Expanding opportunities for professional development may be encouraged by the recognition of its relevance across multiple services and settings, beyond the relatively niche area of palliative care.

Several demographic factors were found to be influential on respondents experiences and needs in regards to professional self-care when working with people with LLCs. Rehabilitation occupational therapists were significantly more likely to report low mood, while those with between 4 and 6 years clinical experience were significantly more likely to experience shock. Age and clinical experience were also found to be influential in regards to the seeking of support from family and friends, and the identification of previous grief and loss, or external professional development. Cipriani et al., [6] also found that young therapists tended to lack knowledge and experience around death and dying, meaning they required greater support to practice professional self-care. These findings suggest that the supports required for occupational therapists to practice effective professional self-care may differ between practice settings and over the course of their career. A standard, organisational wide approach to support may therefore be less effective than one which enables flexibility between individual and group supports, and the content of capacity building initiatives.

Despite the implementation of the multidimensional workplace strategy, just over half of respondents still identified unmet need in regards to professional self-care when working with people with LLCs. The shift in qualitative data to ‘external’ sources of support in the second survey may indicate that the strategy was meeting needs more effectively at the organizational level, but could not meet all of the workforces needs. The findings of this study highlight the significant challenges that evaluation of multi-dimensional interventions for professional self-care pose, given the potential influences of individual lived experience and demographics, support availability, and factors external to the organization itself. A recent systematic review of psychosocial interventions to support staff in palliative care [24] also highlighted the lack of high quality research currently available on this topic, and the need to develop thoughtful interventions which enable professional self-care.

The current lack of evidence around occupational therapy with people with LLCs is a barrier to effective and efficient work with this population [8, 10, 15, 23, 47, 48], however this is beginning to be addressed. The health service has been conducting research and focus groups with senior leaders and staff to develop a broader wellbeing strategy, which will provide a framework and guidance for future initiatives around professional self-care undertaken by occupational therapy. The Occupational Therapy Capabilities Committee has also developed a ‘resilience package’ for implementation across the department, which provides staff with resources designed to support clinicians to implement strategies in the short, medium and longer term. A stronger evidence base around the role of occupational therapy with people with LLCs, (including studies around intervention effectiveness) is also needed to support best care for these clients, and comprehensive education for the clinicians providing them with services.

The multidimensional workplace strategy implemented in this study directly addressed several of the determinants known to increase the effectiveness of allied health knowledge translation [49]. The strategy was aligned with organizational systems (such as supervision) which helped to embed it into practice, and leadership structures at multiple levels of the organization were utilized to drive and maintain momentum, particularly in regards to cultural changes. Professional development provided to the workforce has addressed the need to build capacity for professional self-care with this patient group, while the use of the initial survey to support strategy design took an inclusive approach that included contribution from stakeholders beyond the implementing team. As a result, the findings of this study have provided some evidence in support of the use of a multidimensional strategy to improve the professional self-care of occupational therapists working with people with LLCs.

### Limitations

However, there are several important limitations to this study which impact upon the generalizability of its findings. The mixed methods pre-post methodology does not control for confounding factors, and therefore the findings reported may have also been influenced by other variables. The assumption that departmental change would emerge from the workplace strategy may also have been mistaken, and cannot be demonstrated by the data collected. By categorizing the occupational therapy workforce into acute, rehabilitation, community and aged care cohorts, the findings may not adequately reflect differences within these broad service categories. The survey utilized only provided a limited number of categorical options in response to each question, which the qualitative data indicates may not have been fully inclusive of common experiences and resources. While the health service is large, the study was undertaken within a single service and geographical setting. Finally, analysis of the multidimensional workforce strategy was limited to the occupational therapy workforce, which excludes insights and perspectives from other members of the multidisciplinary team.

## Conclusion

This evaluation of a multidimensional workplace strategy to improve the professional self-care of occupational therapists working with people living with LLCs suggests it made a positive impact on the utilization of supervision, experiences of relevant professional development and perceived organizational culture. This study has also highlighted that occupational therapists working across practice settings, and at different stages of their careers, may have differing needs for support and development around professional self-care when working with people with LLCs.

## Supplementary Information

**Additional file 1: Appendix 1.** Survey Questions.

**Additional file 2.** Non-significant Statistical Findings.

## Data Availability

The datasets used and/or analysed during the current study are available from the corresponding author on reasonable request.

## Abbreviations

LLC: Life Limiting Condition
