# Supplementary material for: Evaluating a multidimensional strategy to improve the professional self-care of occupational therapists working with people with life limiting illness
Source: BMC Palliat Care. 2021 Jan 4;20:2. doi: 10.1186/s12904-020-00695-x (PMC7781397; doi:10.1186/s12904-020-00695-x)
Supplement: Supplementary file 1 — Additional file 1: Appendix 1. Survey Questions. [file 12904_2020_695_MOESM1_ESM.docx]

**Appendix 1 – Survey Questions**

| **Section** | **Question** | **Response Options** |
| --- | --- | --- |
| Demographics | 1. What grade clinician are you? | - *Allied Health Assistant* - *Grade 1* - *Grade 2* - *Senior Clinician* |
| Demographics | 2. How many years clinical experience do you have? | - *Less than 6 mmths (sic)* - *6 mnths (sic) – 1 year* - *1-3 years* - *3-5 years* - *6-10 years* - *> 10 years* |
| Demographics | 3. In which clinical cohort are you currently working? You may select more than one option. | - *Acute* - *Aged Care* - *Community Based Rehabilitation* - *Immediate Response Service* - *Paediatrics* - *Plastics* - *Rehabilitation* - *Other (please specify)* |
| Exposure | 4. Have you ever completed therapy with patients diagnosed with a terminal condition? (Terminal conditions may include: patients made palliative or who have a terminal illness, end-stage chronic conditions, degenerative / neurological conditions, 'life-threatening' or 'life-limiting' illnesses). | - *Yes* - *No* |
| Exposure | 5. Have you completed therapy with patients diagnosed with a terminal condition in the past month? | - *Yes* - *No* |
| Impact | 6. What emotions if any, have you experienced while completing therapy with patients diagnosed with a terminal condition? You may select more than one option. | - *Sadness* - *Anger* - *Shock* - *Helplessness* - *Not applicable* - *Other (please specify)* |
| Impact | 7. Has the experience of completing therapy with patients diagnosed with a terminal illness ever contributed to the following? You may select more than one option. | - *Strain on personal relationships* - *Feeling less motivated to come to work* - *Decreased job satisfaction* - *Lowered mood / decreased well-being* - *Less likely to continue in current clinical area* - *None of the above* - *Other (please specify)* |
| Strategies | 8a. Have you ever received any formal education for dealing with grief/loss or professional self care? | - *Yes* - *No* |
| Strategies | 8b. If yes, when was this complete? | - *During university degree* - *Internal work in-service* - *External professional development course* - *Post graduate study* - *Other (please specify)* |
| Resources | 9. What supports are you aware of for staff dealing with grief/loss or reduced professional self care? This may be within the Occupational Therapy department or more broadly at [health service] | *Open answer* |
| Resources | 10a. Have you ever accessed any service to improve your professional self-care? This may be external to or within Western Health. | - *Yes* - *No* |
| Resources | 10b. If yes, please provide further information about the service accessed and whether it provided adequate support. | *Open answer* |
| Resources | 11. Do you feel confident to access professional self care support within the workplace (e.g. colleagues, supervision, Employee Assistance Program?) | - *Yes* - *No* |
| Resources | 12. What resources or supports do you think are beneficial to assist clinicians in dealing with grief/loss or reduced professional self-care? You may select more than one option. | - *Supervision prompts specific to self awareness/ personal strategies* - *Resilience education/ information* - *Increased awareness of supports* - *Department in-services* - *None of the above* - *Other (please specify)* |
|  | 13. Is there anything more you would like to share with us that you feel would be useful to help us understand your experience of working with patients living with a terminal condition? | *Open answer* |
