# Supplementary material for: Evaluating a multidimensional strategy to improve the professional self-care of occupational therapists working with people with life limiting illness
Source: BMC Palliat Care. 2021 Jan 4;20:2. doi: 10.1186/s12904-020-00695-x (PMC7781397; doi:10.1186/s12904-020-00695-x)
Supplement: Supplementary file 2 — Additional file 2. Non-significant Statistical Findings. [file 12904_2020_695_MOESM2_ESM.docx]

**Additional File 2**

**Non-significant Statistical Findings**

Practice Area

|  |  | n identifying experience | Proportion (%) | Significance |
| --- | --- | --- | --- | --- |
| Sadness | Acute | 23 | 88.5 | H(3) = 0.59  p = 0.90 |
|  | Aged Care | 38 | 95.0 |  |
|  | Community | 0 | 0.0 |  |
|  | Rehabilitation | 0 | 0.0 |  |
| Anger | Acute | 3 | 11.5 | H(3) = 0.28  p = 0.96 |
|  | Aged Care | 3 | 7.5 |  |
|  | Community | 5 | 20.0 |  |
|  | Rehabilitation | 2 | 16.7 |  |
| Shock | Acute | 2 | 7.7 | H(3) = 1.67  p = 0.64 |
|  | Aged Care | 10 | 25.0 |  |
|  | Community | 4 | 16.0 |  |
|  | Rehabilitation | 1 | 8.3 |  |
| Helplessness | Acute | 7 | 26.9 | H(3) = 5.61  p = 0.13 |
|  | Aged Care | 15 | 37.5 |  |
|  | Community | 4 | 16.0 |  |
|  | Rehabilitation | 1 | 8.3 |  |
| Strain on Personal Relationships | Acute | 1 | 3.9 | H(3) = 0.92  p = 0.82 |
|  | Aged Care | 1 | 2.5 |  |
|  | Community | 14 | 56.0 |  |
|  | Rehabilitation | 8 | 66.7 |  |
| Feeling Less Motivated to Come to Work | Acute | 0 | 0.0 | H(3) = 1.12  p = 0.77 |
|  | Aged Care | 5 | 12.5 |  |
|  | Community | 6 | 24.0 |  |
|  | Rehabilitation | 3 | 25.0 |  |
| Decreased Job Satisfaction | Acute | 3 | 11.5 | H(3) = 0.52  p = 0.91 |
|  | Aged Care | 4 | 10.0 |  |
|  | Community | 2 | 8.0 |  |
|  | Rehabilitation | 2 | 16.7 |  |
| Less Like to Continue in Current Role | Acute | 2 | 7.7 | H(3) = 0.29  p = 0.96 |
|  | Aged Care | 2 | 5.0 |  |
|  | Community | 24 | 96.0 |  |
|  | Rehabilitation | 10 | 83.3 |  |

Clinical Experience

|  |  | n identifying experience | Proportion (%) | Significance |
| --- | --- | --- | --- | --- |
| Sadness | 0-3 | 25 | 89.3 | H(3) = 0.24  p = 0.97 |
|  | 3-5 | 19 | 90.5 |  |
|  | 6-10 | 27 | 96.4 |  |
|  | 10+ | 24 | 92.3 |  |
| Anger | 0-3 | 4 | 14.3 | H(3) = 1.53  p = 0.67 |
|  | 3-5 | 4 | 19.1 |  |
|  | 6-10 | 0 | 0.0 |  |
|  | 10+ | 2 | 7.7 |  |
| Helplessness | 0-3 | 13 | 46.4 | H(3) = 1.80  p = 0.61 |
|  | 3-5 | 11 | 52.4 |  |
|  | 6-10 | 11 | 39.3 |  |
|  | 10+ | 9 | 34.6 |  |
| Strain on Personal Relationships | 0-3 | 3 | 10.7 | H(3) = 0.22  p = 0.97 |
|  | 3-5 | 1 | 4.7 |  |
|  | 6-10 | 2 | 7.1 |  |
|  | 10+ | 1 | 3.9 |  |
| Feeling Less Motivated to Come to Work | 0-3 | 5 | 17.9 | H(3) = 0.87  p = 0.83 |
|  | 3-5 | 2 | 9.5 |  |
|  | 6-10 | 2 | 7.1 |  |
|  | 10+ | 1 | 3.9 |  |
| Decreased Job Satisfaction | 0-3 | 5 | 17.9 | H(3) = 0.26  p = 0.97 |
|  | 3-5 | 3 | 14.3 |  |
|  | 6-10 | 3 | 10.7 |  |
|  | 10+ | 3 | 11.5 |  |
| Lowered Mood / Decreased Wellbeing | 0-3 | 10 | 35.7 | H(3) = 2.09  p = 0.55 |
|  | 3-5 | 11 | 52.4 |  |
|  | 6-10 | 13 | 46.4 |  |
|  | 10+ | 8 | 30.8 |  |
| Less Like to Continue in Current Role | 0-3 | 1 | 3.6 | H(3) = 2.08  p = 0.98 |
|  | 3-5 | 0 | 0.0 |  |
|  | 6-10 | 1 | 3.6 |  |
|  | 10+ | 2 | 7.7 |  |

Grade Level

|  |  | n identifying experience | Proportion (%) | Significance |
| --- | --- | --- | --- | --- |
| Sadness | AHA | 8 | 88. 9 | H(3) = 0.74  p = 0.86 |
|  | Grade 1 | 27 | 87.1 |  |
|  | Grade 2 | 44 | 97.8 |  |
|  | Senior Clinician | 16 | 88.9 |  |
| Anger | AHA | 1 | 11.1 | H(3) = 0.23  p = 0.97 |
|  | Grade 1 | 4 | 12.9 |  |
|  | Grade 2 | 3 | 6.7 |  |
|  | Senior Clinician | 2 | 11.1 |  |
| Shock | AHA | 1 | 11.1 | H(3) = 2.08  p = 0.56 |
|  | Grade 1 | 10 | 32.3 |  |
|  | Grade 2 | 8 | 17.8 |  |
|  | Senior Clinician | 2 | 11.1 |  |
| Helplessness | AHA | 3 | 33.3 | H(3) = 1.14  p = 0.77 |
|  | Grade 1 | 16 | 51.6 |  |
|  | Grade 2 | 18 | 40.0 |  |
|  | Senior Clinician | 7 | 38.9 |  |
| Strain on Personal Relationships | AHA | 0 | 0.0 | H(3) = 0.71  p = 0.87 |
|  | Grade 1 | 4 | 12.9 |  |
|  | Grade 2 | 3 | 6.7 |  |
|  | Senior Clinician | 0 | 0.0 |  |
| Feeling Less Motivated to Come to Work | AHA | 1 | 11.1 | H(3) = 1.49  p = 0.68 |
|  | Grade 1 | 6 | 19.4 |  |
|  | Grade 2 | 3 | 6.7 |  |
|  | Senior Clinician | 0 | 0.0 |  |
| Decreased Job Satisfaction | AHA | 0 | 0.0 | H(3) = 0.83  p = 0.84 |
|  | Grade 1 | 6 | 19.4 |  |
|  | Grade 2 | 6 | 13.3 |  |
|  | Senior Clinician | 2 | 11.1 |  |
| Lowered Mood / Decreased Wellbeing | AHA | 2 | 22.2 | H(3) = 1.03  p = 0.79 |
|  | Grade 1 | 13 | 41.9 |  |
|  | Grade 2 | 19 | 42.2 |  |
|  | Senior Clinician | 8 | 44.4 |  |
| Less Likely to Continue in Current Role | AHA | 0 | 0.0 | H(3) = 0.23  p = 0.97 |
|  | Grade 1 | 1 | 3.2 |  |
|  | Grade 2 | 3 | 6.7 |  |
|  | Senior Clinician | 0 | 0.0 |  |

Note: AHA = Allied Health Clinician
